# Anthrax ET activates Rac1 and RTK signaling to induce F-actin reorganization and endothelial permeability

**DOI:** 10.1016/j.isci.2025.113682

**Published:** 2025-10-03

**Authors:** Prashant Jain, Annabel Guichard, Mahtab Moayeri, Saluja Kaduwal, Margot Mel de Fontenay, Ian Rousseau, Stephen H. Leppla, Ethan Bier

**Affiliations:** 1 Section of Cell and Developmental Biology, UCSD, La Jolla, CA 92093-0335, USA; 2 Tata Institute for Genetics and Society-UCSD, La Jolla, CA 92093-0335, USA; 3 Microbial Pathogenesis Section, Laboratory of Parasitic Diseases, National Institute for Allergy and Infectious Disease, NIH, Bethesda, MD 20892-3202, USA

**Keywords:** Natural sciences, Biological sciences, Biochemistry, Cell biology

## Abstract

Endothelial permeability induced by the potent adenylate cyclase edema toxin (ET) is central to bacterial dissemination and lethal vascular collapse during infections caused by *Bacillus anthracis*. Antibiotic and antitoxin treatments are ineffective against anthrax lethal toxemia once high doses of toxins have been released. This study uncovers a critical cAMP-dependent disruption of the F-actin network by ET in human brain microvascular endothelial cells (HBMECs), mediated by Rac1 and cofilin. Rac1 activation by ET leads to a loss of cell area and monolayer permeability. These effects are preceded by the rapid cAMP-independent activation of IGF1R and EGFR and their respective downstream effectors PI3K/AKT and MEK/ERK, which contribute to F-actin remodeling and permeability induced by ET. Consistent with these findings, Rac1, PI3K, and MEK inhibitors prevent ET-induced edema in a mouse footpad model, providing the *in vivo* pre-clinical validation of their therapeutic potential.

## Highlights

• Anthrax edema toxin (ET) causes F-actin reorganization in human endothelial cells
• cAMP-dependent actin remodeling and monolayer permeability by ET are mediated by Rac1
• cAMP-independent activation of IGF1R and EGFR by ET is necessary for F-actin reorganization
• Rac1, PI3K, and MEK1 inhibitors alleviate ET-induced edema in a mouse footpad assay

## Graphical abstract

**Figure:**
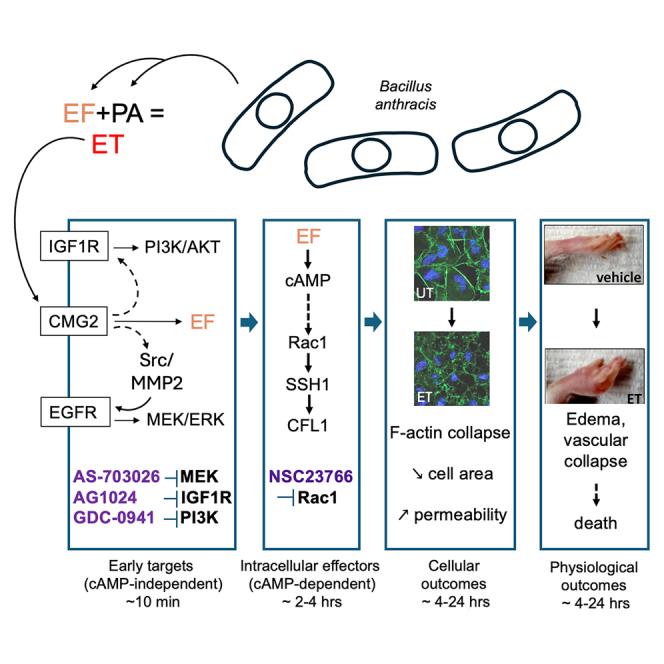

## Introduction

*Bacillus anthracis* (*B.a*), the causative agent of anthrax, holds a special place in the history of microbiology, since the first demonstration in 1876 by Robert Koch of a microorganism producing a lethal infection,^1^ and the iconic public demonstration of a protective vaccine at Pouilly-le-Fort by Louis Pasteur in 1881.^2^ As highlighted by the 2001 biological attacks, *B.a* can be easily weaponized in aerosolized spores and is listed as a Category A Priority Pathogen for biodefense.^3^ Regular anthrax outbreaks are still reported, affecting wildlife, cattle, and occasionally humans, predominantly in developing countries, representing a substantial veterinary and public health burden.^4^^,^^5^ Antibiotic-resistant *B.a* strains have been reported in the wild,^6^ and can be easily selected in the laboratory,^7^ raising additional concerns. Anthrax mortality rates are higher than for most bacterial infections, and depending on the route of infection (cutaneous, gastrointestinal, inhalational, or injectional), range from 20% to 80%. Fatality strongly correlates with bacteremia and toxemia.^8^^,^^9^^,^^10^^,^^11^ Patients presenting late-stage systemic infections endure massive fluid loss resulting in hypovolemic shock, followed by lethal cardiovascular collapse.^12^^,^^13^^,^^14^ The treatment of systemic anthrax infections involves immediate antimicrobial therapy combined with antitoxins (anthrax-specific immunoglobulins), and aggressive fluid drainage with assisted breathing during later stages of infection.^10^^,^^15^ These measures, however, offer very limited protection once the released toxins enter host tissues and cells to subvert vital cellular processes and barrier integrity, causing immune system paralysis and cardiovascular collapse. Therapeutic measures that specifically mitigate the consequences of active cellular intoxication by anthrax toxins are therefore needed to protect patients undergoing late fulminant stage systemic anthrax infection.

Fluid loss during systemic anthrax infection occurs through the degradation of endothelial barrier integrity mediated by the concerted action of two cooperating exotoxins secreted by *B.a*: Edema Toxin (ET) and Lethal Toxin (LT), which enter host cells after binding to either of two surface receptors TEM8 and CMG2.^12^^,^^13^^,^^16^^,^^17^ ET, comprised of two proteins: Protective Antigen (PA), a receptor binding component, and Edema Factor (EF), is a highly active adenylate cyclase,^18^ while LT, composed of Lethal Factor (LF) and PA, is a metalloprotease that cleaves and inactivates most MAPKK to inhibit all branches of MAPK signaling.^19^ While both anthrax toxins have the ability to impair endothelial integrity^14^^,^^20^^,^^21^ and are lethal in a murine model, ET kills mice at lower doses relative to LT.^16^ Previously, we reported that ET blocks exocyst-dependent junctional trafficking to weaken adherens junctions and induce vascular leakage^22^^,^^23^ using a combination of genetic and cell-based mechanistic investigations in *Drosophila* and in a human brain microvascular endothelial cell (HBMEC) model. These and subsequent studies revealed that ET acts through a mechanism involving predominantly the cAMP effectors Epac and Rap1.^22^ Similarly, we found that cAMP-inducing cholera toxin (Ctx) stimulates paracellular ion flow and fluid loss, a process enhanced by the inhibition of Rab11-mediated junctional trafficking.^24^

Here, we report an additional activity of ET, which disrupts the F-actin network in a cAMP-dependent fashion, via the small GTPase Rac1, the phosphatase Slingshot (SSH1), and the actin filament severing protein Cofilin (CFL1), to induce cellular architecture collapse and monolayer permeability. Surprisingly, these effects are also preceded by the cAMP-independent activation of both the insulin growth factor 1 receptor (IGF1R) and epidermal growth factor receptor (EGFR) and their respective signaling pathways. We show that such receptor tyrosine kinase (RTK) signaling contributes to subsequent cAMP-dependent ET-induced actin remodeling. Furthermore, we identify several therapeutic compounds inhibiting both early and late signaling mediators such as IGF1R (AG1024), PI3K (AS-703026), MEK (GDC-0941) and Rac1 (NSC23766) that effectively protect against cellular and physiological consequences of ET toxicity in HBMECs and an *in vivo* mouse challenge model.

## Results

### Anthrax edema toxin induces cortical actin rearrangement in human brain microvascular endothelial cells

The present study investigates the involvement and mechanism of actin cytoskeleton dynamics in ET-induced endothelial barrier dysfunction. The cortical F-actin network provides a structural framework essential for cell shape maintenance and tissue integrity, as well as anchors to junctional components at points of cell-to-cell contacts.^25^^,^^26^^,^^27^^,^^28^ Additionally, these junctional components rely on radial actin cables for vesicular transport to cell-cell junctions.^29^ These considerations place the F-actin network at the heart of endothelial barrier integrity.

**Figure 1:**
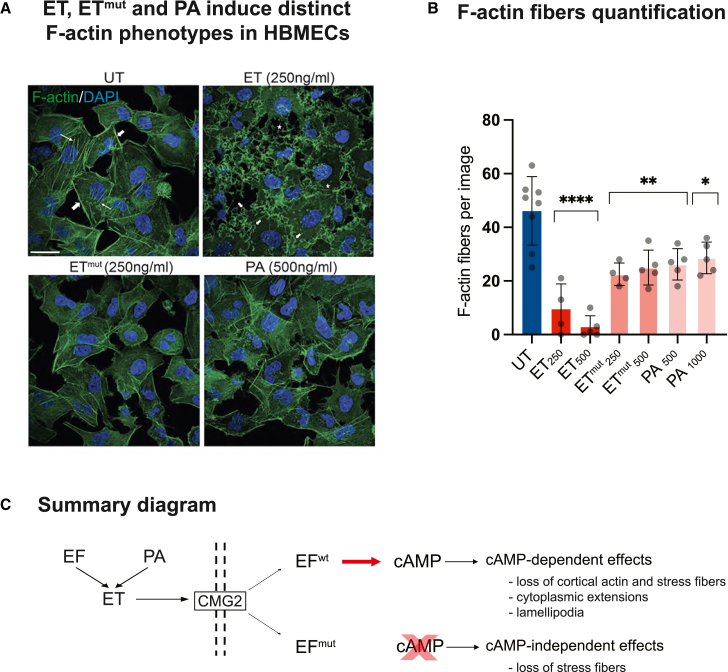
Anthrax ET induces cortical F-actin rearrangement in human brain microvascular endothelial cells (HBMECs) (A) HBMECs were treated with 250 ng/mL EF + 500 ng/ml PA or catalytically dead ET^mut^ (EF K313R + PA, or PA alone, 500 ng/mL) for 4 h and stained with Alexa Fluor 488 conjugated phalloidin to visualize F-actin. For this and subsequent figures, only the concentration of EF is indicated, while the concentration of PA is double that of EF. Untreated cells show cortical actin (thick arrows) and stress fibers (thin arrows). ET treatment causes a profound disruption of the F-actin network, with a loss of cortical actin and stress fibers, and induces the formation of membrane ruffling (short arrows) and thin cytoplasmic extensions (stars). ET^mut^ and PA have milder phenotypes, namely a loss of cortical F-actin and stress fibers. Scale bar represents 30 μm. Data are representative of 2 independent experiments. (B) Actin fiber quantifications for representative images in panel A. Untreated cells display numerous F-actin fibers, which are eradicated in ET-treated cells in a dose-dependent manner, while ET^mut^ and PA only reduce the number of fibers. Statistical significance was evaluated using unpaired Student’s t test and represented using standard symbolism (∗∗∗∗*p* < 0.0001, ∗∗*p* < 0.01, ∗*p* < 0.05). Error bars represent standard deviation. (C) Summary diagram represents edema toxin (ET = EF + PA) interacting with the CMG2 surface receptor for cell entry and producing cAMP-dependent (ET) and cAMP-independent (ET^mut^ and PA) F-actin phenotypes. See also Figure S1.

We evaluated changes in the actin cytoskeleton in response to a 4h ET treatment in human brain microvascular endothelial cells (HBMECs), a common cell line for blood brain barrier studies,^30^ which is also known for its sensitivity to anthrax toxins.^21^ ET induced severe defects in a dose-dependent fashion, including loss of cortical actin and stress fibers, as well as F-actin clustering and lamellipodia (Figure 1A). These changes were accompanied by striking changes in cellular morphology, including the formation of long cytoplasmic extensions. Interestingly, the catalytically dead ET mutant (ET^mut^ = EF^K313R^ + PA), which exhibits a 10,000-fold reduction in adenylate cyclase activity relative to wild-type ET^31^ and PA alone, also caused F-actin remodeling, mainly characterized by a loss of stress fibers (see Figures 1A, 1B, and S1, which provide image-based quantifications of cortical actin and stress fibers, and Figure S2, which provides intensity-based quantifications of ET^mut^ and PA-induced phenotypes), although this effect was substantially weaker than that observed with wild-type cAMP-stimulating ET, and did not display significant dose-dependence (Figures 1B, S1, and S2). These results indicate that ET disrupts F-actin dynamics predominantly in a cAMP-dependent fashion, although a portion of these effects can be attributed to a cAMP-independent activity (Figure 1C).

### Edema toxin activates Rac1 signaling to disrupt F-actin organization

**Figure 2:**
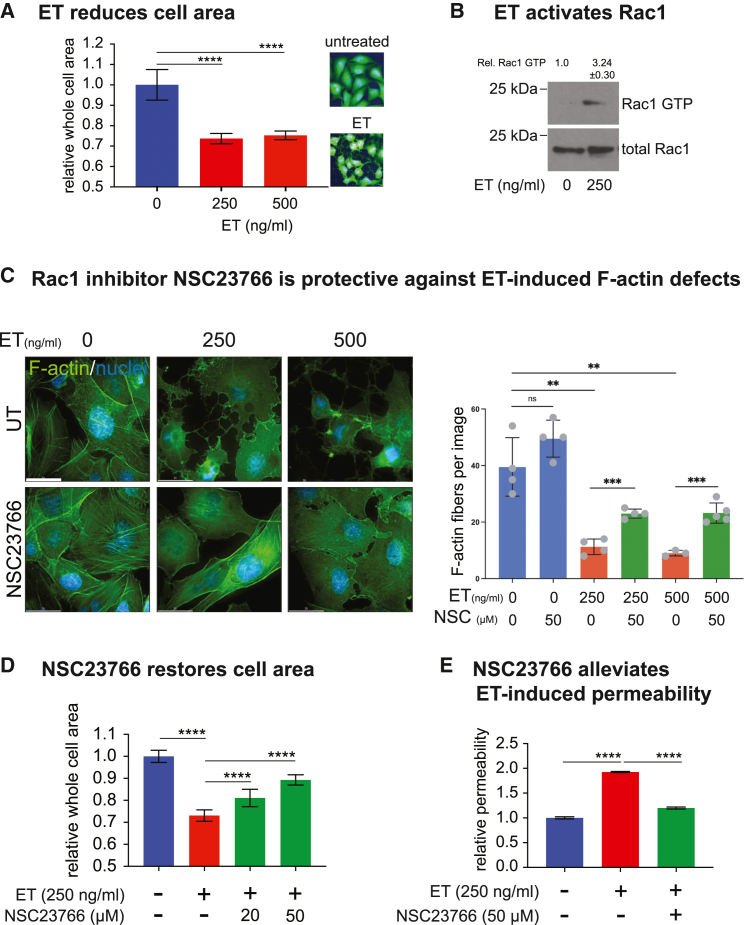
ET-induced F-actin rearrangement is dependent on Rac1 activation (A) Quantification of “whole-cell area” of HBMECs stained with HCS CellMask Green to visualize the cytoplasm from 12 wells for each treatment (total cell number = 13,000) using an automated image analysis software. ET treatment for 4 h induced a significant reduction in whole-cell area (inset = representative picture). Data is representative of 3 independent experiments. (B) Whole-cell lysates of HBMEC were incubated with PAK-PBD beads, which specifically bind to GTP-bound Rac1. Bead-bound Rac1-GTP and total Rac1 levels in the cell lysates were determined by western blot with anti-Rac1 antibody. The mean relative pixel intensities (±S.D.) of Rac1-GTP versus total Rac1 from 2 independent experiments are indicated. The relative Rac1-GTP levels are ∼3 times higher in ET treated cells. (C) HBMECs were treated with 250 or 500 ng/mL ET for 4 h, in the presence or absence of the Rac1 inhibitor NSC23766 (50 μM) and stained with Alexa Fluor 488 Phalloidin to visualize F-actin (left panels). Rac1 inhibition almost completely inhibited ET-induced F-actin rearrangement. Scale bar represents 30 μm. F-actin fiber quantifications show that NSC23766 significantly rescues ET-induced defects (right panel). (D) Medium-throughput of whole-cell area quantifications of HBMECs, treated with ET (250 ng/mL) for 4 h, in the presence or absence of the Rac1 inhibitor NSC23766 (50 μM) at the indicated concentrations. Whole-cell area was quantified from 8 wells for each treatment using automated image analysis software (total cell number>10,000). Rac1 inhibition significantly rescued ET-induced whole-cell area reduction in a dose-dependent manner. (E) HBMECs were grown to full confluence in transwell inserts and treated with ET (250 ng/mL) for 24 h, in the presence or absence of the Rac1 inhibitor NSC23766 (50 μM). Change in monolayer permeability was assessed by measuring the diffusion of Evans blue dye from the apical to the basal chamber of transwell inserts. ET significantly increased monolayer permeability, which was rescued by NSC23766. For panels A, C, D and E, statistical significance was evaluated using unpaired Student’s t test and represented using standard symbolism (∗∗∗∗*p* < 0.0001, ∗∗∗*p* < 0.001, ∗∗*p* < 0.01). Error bars represent standard deviation. See also Figures S2 and S3.

We first examined the cAMP-dependent activity of ET. Using the CellMask Green dye that permits the quantitative evaluation of whole cell area, we found that ET treatment produced a 2-fold reduction in cell area (Figure 2A), These substantial changes in cellular morphology, however, were not associated with a loss in cell viability at the toxin concentrations tested (Figure S3A).

We investigated the molecular mechanisms involved in ET-induced actin remodeling and cell surface reduction by evaluating the contributions of Rac1 and RhoA, two small GTPases playing major roles in regulating actin dynamics.^32^^,^^33^^,^^34^ These GTPases are activated upon GDP to GTP exchange mediated by specific GEFs (guanine nucleotide exchange factors). The GTP-bound activated proteins then act on various effectors to regulate different aspects of actin dynamics, including stabilization, polymerization, and cross-linking.^35^^,^^36^ In lysates from HBMECs treated with ET for 4 h, we observed a substantial (∼3-fold) increase in Rac1-GTP (active Rac1) levels (Figure 2B), while total levels of Rac1 remained unchanged. On the other hand, RhoA-GTP levels did not increase in ET-treated cells compared to untreated cells and were even reduced at higher toxin concentrations (Figure S3B). Moreover, HBMECs treated with ET showed greater degrees of F-actin rearrangement in the presence of Y27632, a specific inhibitor of Rho-associated protein kinase (ROCK), a downstream effector of RhoA (Figure S3C), suggesting that Rho plays an inhibitory role in ET-induced actin remodeling. In contrast, pharmacological inhibition of Rac1 with the specific inhibitor NSC23766 potently rescued ET-mediated actin rearrangements (Figure 2C) and significantly attenuated the reduction of whole-cell area in HBMEC cultures in a dose-dependent manner (Figure 2D). These data indicate that ET-induced actin rearrangements are mediated primarily through Rac1 activity, acting in opposition to RhoA.

Next, we examined the relationship between ET-induced actin rearrangements and cell-cell barrier disruption by evaluating the permeability of HBMEC monolayers following ET treatment in the absence or presence of the Rac1 inhibitor (50 μM). Using a colorimetric transwell assay,^23^ we found that ET induced a significant increase in HBMEC monolayer permeability within 24h of treatment. Consistent with its mitigating effect on F-actin rearrangement, the Rac1 inhibitor also largely reversed ET-dependent barrier permeabilization (Figure 2E).

### Edema toxin induces the activation of the actin severing protein cofilin (CFL1)

**Figure 3:**
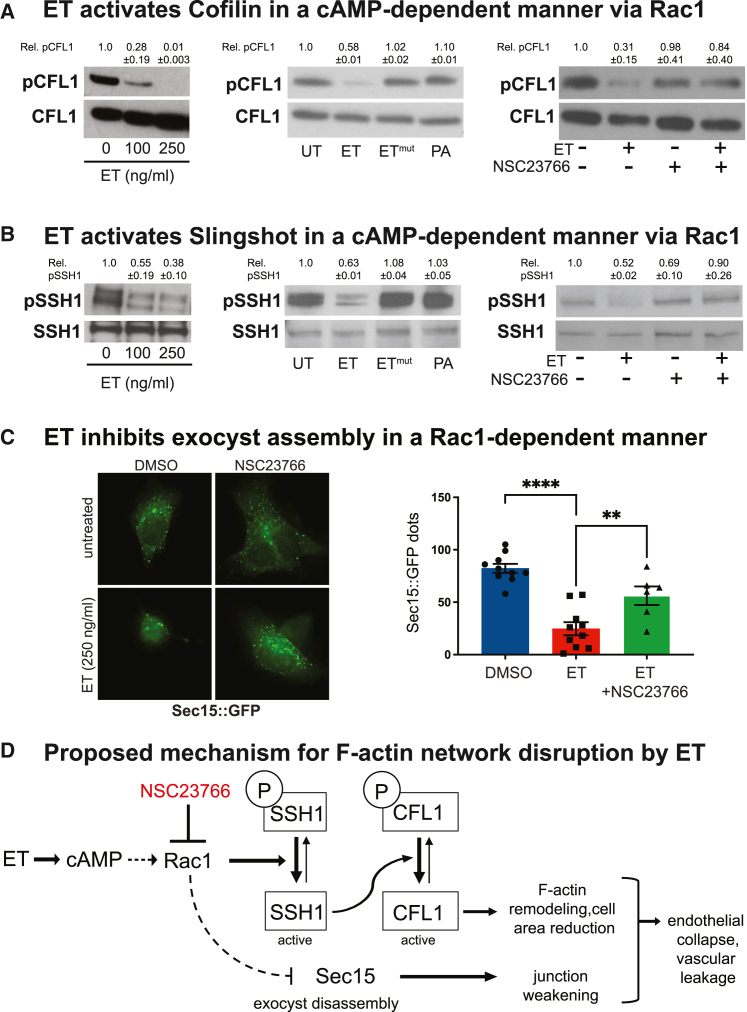
ET induces the activation of the actin severing protein cofilin (CFL1) and Slingshot phosphatase (SSH1) in a Rac1-dependent fashion (A) Activation of CFL1 in HBMECs was assessed by monitoring the levels of CFL1 phosphorylated at Serine 3 relative to total CFL1 in whole-cell lysates by western blotting (WB). Left panel: Phospho-CFL1 levels were reduced by ET in a dose-dependent manner after 4 h of treatment. Middle panel: HBMECs were treated with 250 ng/mL ET, ET^mut^, or 500 ng/mL PA alone for 4 h. Only the catalytically active ET induced a clear reduction in CFL1 phosphorylation. Right panel: HBMECs were treated with 250 ng/mL ET for 4 h, in the presence or absence of the Rac1 inhibitor NSC23766 (50 μM). NSC23766 suppresses ET-induced CFL1 dephosphorylation. These experiments were performed 2 times. (B) Activation of SSH1 in ET-treated and control cells was assessed by monitoring the levels of SSH1 phosphorylated at Serine 978 in HBMECs by western blot. Left panel: pSSH1 levels were reduced in ET-treated cells. Middle panel: HBMECs were treated with 250 ng/mL ET or ET^mut^, or 500 ng/mL PA for 4 h. Only the catalytically active ET reduced SSH1 phosphorylation. Right panel: HBMECs were treated with 250 ng/mL ET for 4 h, in the presence or absence of the Rac1 inhibitor NSC23766 (50 μM). NSC23766 suppresses ET-induced SSH1 dephosphorylation. These experiments were performed 2 times. (C) Left panel: HBMECs were transfected with a Sec15-GFP-expressing plasmid. Over-expressed Sec15-GFP assembles in numerous spherical cytoplasmic structures. ET treatment (250 ng/mL) prevented assembly of Sec15-GFP complexes, and this phenotype was rescued by Rac1 inhibitor NSC23766 (50 μM). Right panel: Quantification of the Sec15-GFP dots shown in the left panel. Statistical significance was evaluated using unpaired Student’s t test and represented using standard symbolism (∗∗∗∗*p* < 0.0001, ∗∗*p* < 0.01). Error bars represent standard deviation. (D) Proposed model for cAMP-dependent effects on F-actin remodeling by ET. After cell entry, EF produces pathological levels of cAMP, causing the activation of Rac1, dephosphorylation of SSH1, and subsequent dephosphorylation (activation) of CFL1. Activated CFL1 inhibits actin polymerization and promotes actin severing, altering cell shape, and reducing cell area. ET also inhibits the assembly of the Sec15-containing exocyst in a Rac1-dependent fashion. The exocyst is involved in trafficking Cadherins to intercellular junctions, a process blocked by ET. See also Figure S4.

Rac1 impinges on actin polymerization dynamics by modulating the activity of actin-severing proteins such as cofilin (CFL1). The actin-depolymerizing function of CFL1 is neutralized by phosphorylation at Ser-3.^37^ We examined the potential role of phospho-CFL1 (pCFL1) using an antibody that specifically recognizes this phosphorylation event and observed a profound reduction in pCFL1 levels in HBMECs after 4 h of ET treatment, indicating a toxin-dependent activation of CFL1 (Figure 3A, left panel). This effect was clearly cAMP-dependent as neither catalytically inactive ET mutant (ET^mut^ = EF^K313R^ + PA), nor PA alone, induced the dephosphorylation of pCFL1 (Figure 3A, middle panel). We analyzed the ET-dependent activation of CFL1 further by investigating the role of Rac1 in this process: Rac1 inhibitor NSC23766 almost completely reverted the ET-induced dephosphorylation of pCFL1 in HBMEC (Figure 3A, right panel), consistent with Rac1 mediating CFL1 activation by ET.

We also examined the potential role of Slingshot Homolog 1 (SSH1) in the ET-dependent activation of CFL1, since this phosphatase is responsible for the selective dephosphorylation of pCFL1 at Ser-3.^38^ SSH1 is activated in a Rac1-dependent manner^39^^,^^40^^,^^41^^,^^42^^,^^43^ and is negatively regulated by phosphorylation at multiple serine residues, including Serine 978.^44^^,^^45^^,^^46^ We observed a strong decrease in pSSH1 (Ser-978) levels in HBMECs treated with ET for 4 h relative to untreated control cells (Figure 3B, left panel) suggesting that ET induces SSH1 activation via the dephosphorylation of pSSH1 at Ser-978 and potentially at other residues. In agreement with the requirement of cAMP production by EF for CFL1 activation, neither ET^mut^, nor PA alone induced the dephosphorylation of pSSH1 (Figure 3B, middle panel). Additionally, the reduction of pSSH1 levels by ET was effectively rescued by the Rac1 inhibitor NSC23766 (Figure 3B, right panel), indicating that ET induces SSH1 activation in a Rac1-dependent manner. Of note, the dephosphorylation of pSSH1 was observed between 2 and 4 h after ET treatment, but not at earlier time periods (Figure S4), consistent with the cAMP-dependence of SSH1 dephosphorylation.

### Rac1 activity contributes to edema toxin inhibition of exocyst-dependent trafficking

Our previous studies revealed that ET disrupts the exocyst-mediated trafficking of junctional components such as cadherins to intercellular junctions.^23^ Abnormal activation of the cAMP effector EPAC indirectly prevents assembly of the exocyst, a Sec15-rich multi-protein complex that allows the fusion of cargo vesicles at apical sites of the plasma membrane.^22^ We wondered whether the actin remodeling activity of ET characterized here might also contribute to this process. Typically, Sec15-GFP over-expression results in the formation of large grape-like GFP-positive aggregates,^23^^,^^47^ which are abolished by ET.^22^^,^^23^ We examined whether this effect of ET was dependent on Rac1, using the specific inhibitor NSC23766. We observed that the inhibition of Sec15-GFP aggregates by ET was significantly rescued by NSC23766, while HBMECs treated with ET alone showed the expected reduction in Sec15-GFP aggregates and diffuse fluorescence (Figure 3C). These results suggest that Rac1 mediates -at least in part- the inhibition exerted by ET on exocyst assembly. Altogether, our findings indicate that the dephosphorylation of CFL1, SSH1 activation, and the inhibition of exocyst assembly occur downstream of Rac1 activation by ET in a cAMP-dependent manner, resulting in actin remodeling, cell surface reduction, and increased monolayer permeability (see Figure 3D for summary diagram).

### Edema toxin activates tyrosine kinase receptors type 1 insulin growth factor receptor and epidermal growth factor receptor in a cAMP-independent manner

**Figure 4:**
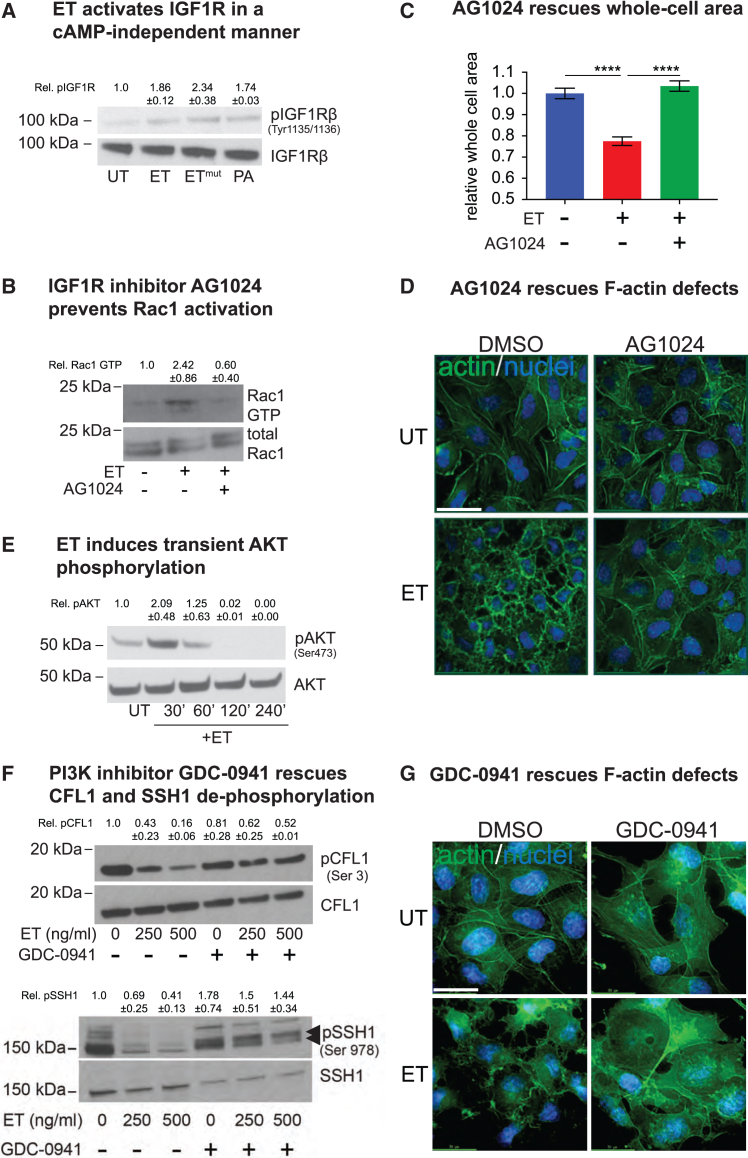
Early IGF1R activation by ET contributes to toxicity (A) IGF1R activation in HBMECs was assessed by monitoring the levels of IGF1R phosphorylated at tyrosine 1135 and 1136 relative to total IGF1R by western blot. IGF1R activation was observed following 250 ng/ml treatment with ET, ET^mut^ or 500 ng/ml PA within 10 min of treatment. (B) HBMECs were treated with 250 ng/mL ET for 4 h, in the presence or absence of the IGF1R inhibitor AG1024. Whole-cell lysates were incubated with PAK-PBD beads. Bead bound Rac1-GTP and total Rac1 levels in cell lysate were determined by WB with anti-Rac1 antibody. IGF1R inhibition decreased Rac1-GTP levels in ET treated cells. (C) Quantification of whole-cell area of HBMECs treated with ET (250 ng/mL) for 4 h, in the presence or absence of the IGF1R inhibitor AG1024. Cells were fixed and stained with HCS CellMask Green to visualize the cytoplasm. Whole-cell area was quantified from 8 wells for each treatment (n = Total cell number) using automated image analysis software. IGF1R inhibition completely rescued ET-induced reduction in whole-cell area. Statistical significance was evaluated using an unpaired Student t test and represented using standard symbolism (^∗∗∗∗^p < 0.0001). Error bars indicate standard deviation. (D) HBMECs were treated with 250 ng/mL ET for 4 h, in the presence or absence of the IGF1R inhibitor AG1024 and stained with Alexa Fluor 568-Phalloidin to visualize F-actin. AG1024 blocked ET-induced F-actin rearrangement. Scale bar represents 30 μm. (E) Activation of downstream IGF1R effector AKT was assessed using a phospho-specific antibody (anti-phospho-Ser-473 AKT) in HBMECs treated with 250 ng/mL ET for the indicated time periods. Total and phosphorylated AKT levels were determined by WB. ET induced transient phosphorylation of AKT within 30 min of treatment. (F) Activation of CFL1 and SSH1 was assessed by monitoring the levels of pCFL1 (Ser3) and pSSH1 (Ser978) in whole-cell lysates by WB. PI3K inhibitor GDC-0941 prevented CFL and SSH1 dephosphorylation induced by a 4 h ET treatment (250 ng/ml and 500 ng/ml). Double arrow indicates pSSH1 in the lower panel. (G) HBMECs were treated with 250 ng/mL ET for 4 h, in the presence or absence of the PI3K inhibitor GDC-0941, and stained with Alexa Fluor 568 conjugated phalloidin to visualize F-actin. GDC-0941 blocks ET-induced cortical actin rearrangement. Scale bar represents 30 μm. These experiments (A–G) were performed 2 times.

In a previous report, we showed that AG1024, a specific inhibitor of type 1 insulin growth factor receptor (IGF1R) autophosphorylation,^48^^,^^49^ strongly mitigated ET-induced edema in an *in vivo* mouse footpad assay,^22^ suggesting a possible role of IGF1R in mediating ET-induced edema. IGF1R, a receptor tyrosine kinase (RTK), undergoes autophosphorylation at tyrosine residues 1131, 1135, or 1136 following activation, which triggers downstream signaling via PI3K and AKT to influence a multitude of cellular responses, including proliferation, motility, and adhesion.^50^^,^^51^ We found that HBMECs treated with ET displayed a noticeable increase in the tyrosine phosphorylation of IGF1R at Tyr-1135/1136 (Figure 4A). Importantly, this effect was visible early, within 10 min of intoxication, in contrast to SSH1 phosphorylation, which was evident only at the 4 h time point (Figure S4). This rapid kinetics suggests that the IGF1R mediated activity of ET could be cAMP independent. Consistent with this hypothesis, the tyrosine phosphorylation of IGF1R was also observed in HBMECs treated with catalytically inactive ET^mut^ (EF K313R + PA), as well as with PA alone, indicating that cAMP production is not a prerequisite for IGF1R activation (Figure 4A), which rather may depend on the binding of PA with a surface component of host cells. We evaluated the role of IGF1R in ET intoxication using chemical inhibition of IGF1R autophosphorylation (with AG1024) and found that it rendered HBMECs almost completely refractory to the effects of ET (4 h treatment) with respect to Rac1 activation (Figure 4B), cell area reduction (Figure 4C), and F-actin rearrangement (Figure 4D). These unexpected observations indicate that rapid cAMP-independent IGF1R activation by ET contributes to subsequent Rac1-dependent outcomes. IGF1R signaling involves the activation of downstream components PI3K and AKT. Consistent with the activation of IGF1R by ET, we also observed strong transient activation of its downstream effector AKT within 30 min of toxin exposure (Figure 4E).

In line with these obervations, PI3K inhibitor GDC-0941 prevented ET-induced CFL1 and SSH1 activation, as well as F-actin rearrangements (Figures 4F–4G), indicating that this key IGF1R signaling effector is important in mediating ET-dependent cytoskeletal alterations.

**Figure 5:**
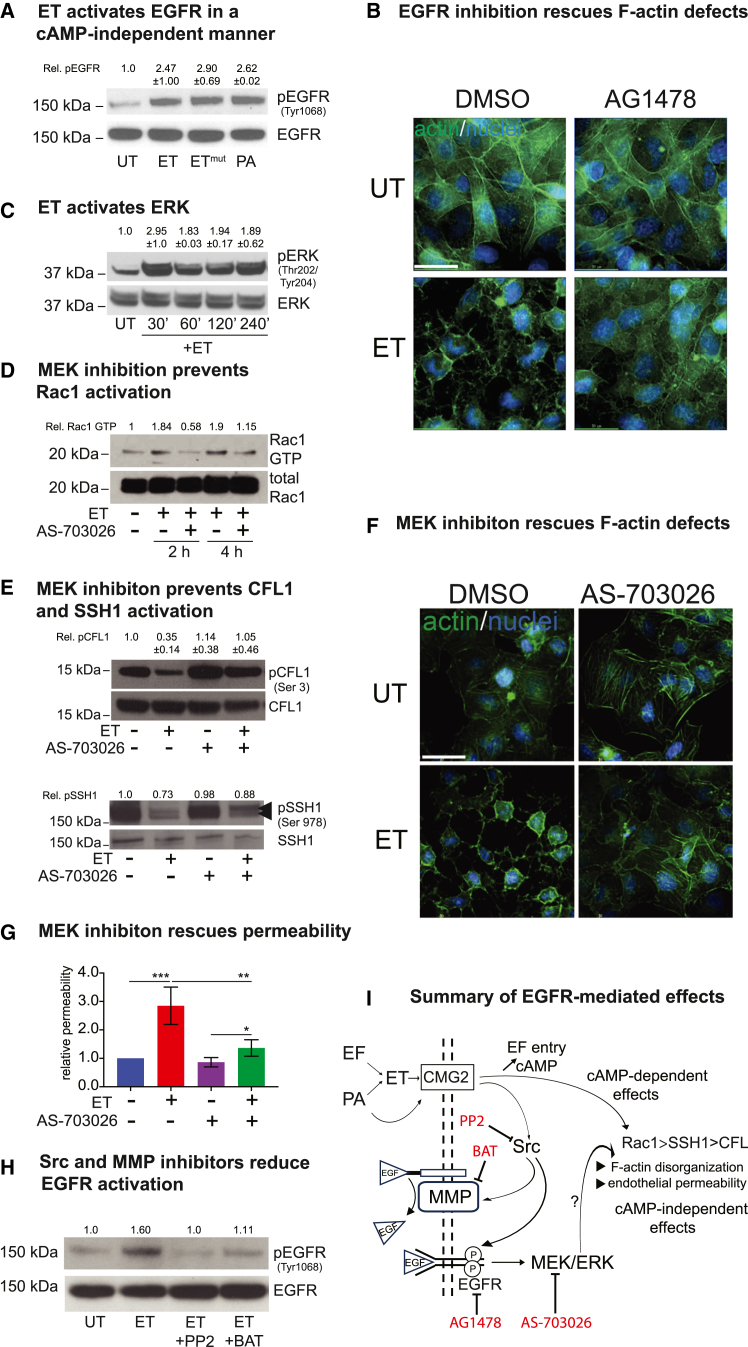
Early EGFR activation by ET contributes to toxicity (A) EGFR activation in HBMECs was assessed by monitoring the levels of phosphorylated Tyr1068 by WB. Robust EGFR activation was observed following treatment with 250 ng/mL ET, ET^mut^, or 500 ng/ml PA within 10 min of treatment. (B) HBMECs were treated with 250 ng/mL ET for 4 h in the presence or absence of the EGFR inhibitor AG1478 and stained with Alexa Fluor 568 conjugated phalloidin to visualize F-actin. EGFR inhibition considerably reduced ET-induced cortical actin rearrangement. Scale bar represents 30 μm. (C) ERK activation at different time points was monitored by WB using a phospho-specific antibody. Robust and sustained ERK activation was observed after 30 min of ET treatment. (D) Whole-cell lysates were incubated with PAK-PBD beads, which specifically bind to active GTP-bound Rac1. Bead-bound Rac1-GTP and total Rac1 levels were determined by WB with anti-Rac1 antibody. Pharmacological inhibition of MEK decreased the relative levels of Rac1-GTP in ET (250 ng/mL) treated cells (2 h and 4h), as revealed by 3 experimental repeats. (E) HBMECs were treated with 250 ng/mL ET for 4 h, in the presence or absence of the MEK inhibitor AS-703026 (50 μM). Pharmacological inhibition of MEK prevents CFL1 and SSH1 dephosphorylation in ET treated cells. Double arrow indicates pSSH1 in the lower panel. (F) MEK inhibition with AS-703026 (50 μM) rescued F-actin remodeling by ET (250 ng/mL, 4 h) visualized with Alexa Fluor 568 conjugated phalloidin. Scale bar represents 30 μm. (G) HBMECs were grown to confluence in transwell inserts and treated with ET (250 ng/mL) for 24 h, in the presence or absence of MEK inhibitor AS-703026 (50 μM). Change in monolayer permeability was assessed by assaying the diffusion of Evans blue dye. MEK inhibition significantly reduced ET-induced permeability. Statistical significance was evaluated using unpaired Student t test and represented using standard symbolism (^∗∗∗^p < 0.001, ^∗∗^p < 0.01, ^∗^p < 0.05). Error bars indicate standard deviation. (H) HBMECs were treated with 250 ng/mL ET in the presence or absence of Src inhibitor PP2, and MMP inhibitor Batimastat (BAT). Both PP2 and Batimastat reduced the levels of EGFR phosphorylation induced by ET. (I) Summary diagram representing possible mechanisms for EGFR activation by ET, and the contribution of EGFR signaling to ET-induced F-actin defects.

Because other RTKs, such as EGFR, have been implicated in modulating actin dynamics,^52^^,^^53^^,^^54^^,^^55^ and are activated by several bacterial pathogens,^56^^,^^57^^,^^58^^,^^59^ we examined the possibility that EGFR also might be recruited by ET to promote actin remodeling. We found that, similarly to IGF1R, EGFR phosphorylation (at Tyr1068) was robustly induced by ET, as well as by its catalytically inactive form ET^mut^, or PA alone within 10 min of intoxication (Figure 5A). Consistent with a role for EGFR activation in ET-induced actin remodeling, the inhibition of EGFR with AG1478 rescued the F-actin defects produced by ET (Figure 5B). ET treatment also stimulated ERK phosphorylation in a rapid (less than 30 min) and sustained manner (Figure 5C). Consistent with these observations, the inhibition of the downstream EGFR signaling component MEK (with AS-703026) prevented ET-induced Rac1 activation (Figure 5D), CFL1 and SSH1 de-phosphorylation (Figure 5E), F-actin network defects (Figure 5F), and elevated cell permeability (Figure 5G). Altogether, these results indicate that the stimulation of EGFR signaling by ET is an integral element of the pathway leading to actin remodeling via the downstream Rac1/CFL1 axis.

We next considered possible mechanisms underlying the observed ET- and PA-induced EGFR activation in HBMECs. Earlier studies have demonstrated the activation of RTK signaling by pathogen effectors,^56^^,^^58^ which often involve the shedding of membrane-bound ligand (e.g., EGF) by cellular metalloproteases (MMPs),^60^^,^^61^^,^^62^ and result in the activation of the cognate receptor. Alternatively, the toxin-induced activation of Src kinases has been reported to directly trigger the phosphorylation of RTKs.^63^ Interestingly, anthrax PA, the binding component of ET holotoxin, has been shown to activate Src kinases in HeLa cells on its own.^64^ We therefore investigated the role of Src kinase and MMP in ET-dependent RTK activation. We observed a robust reduction in ET-induced EGFR phosphorylation in HBMECs treated with ET in the presence of the Src kinase inhibitor PP2 and MMP inhibitor Batismatat (BAT), relative to cells treated with ET alone (Figure 5H). These results indicate that EGFR activation by ET involves both Src kinase and MMP-mediated ligand shedding, suggesting that two parallel mechanisms underlie the rapid cAMP-independent activities of ET (see Figure 5I for summary diagram).

### Pharmacological inhibition of PI3K, MEK, and Rac1 function protects against edema toxin-induced edema *in vivo*

**Figure 6:**
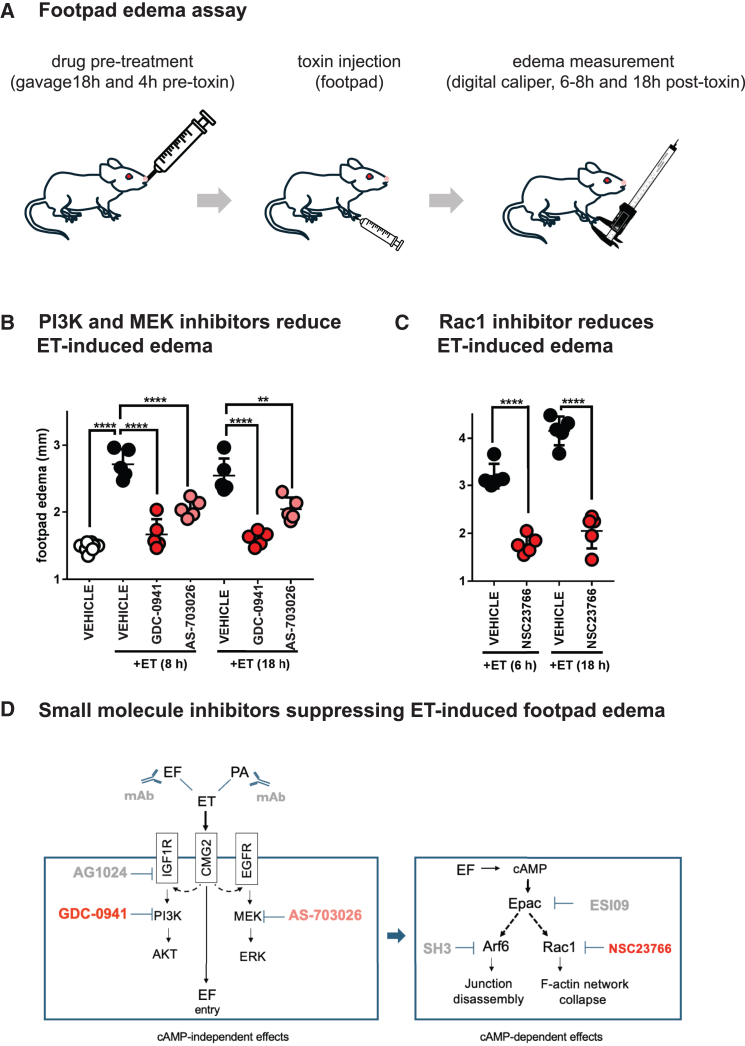
Pharmacological inhibition of PI3K, MEK, and Rac1 protects against ET-induced edema *in vivo* (A) *In vivo* ET-induced edema experimental procedure involving drug pre-treatment by gavage, ET footpad injection, and edema measurement with a digital caliper. (B) Pre-treatment with PI3K inhibitor GDC-0941 (75 mg/kg) or MEK inhibitor AS-703026 (33 mg/kg), significantly reduced the footpad edema measured at the indicated time periods following ET injection (0.15 μg ET/paw). (C) Co-treatment with Rac1 inhibitor NSC23766 (10 mg/kg) significantly reduces the footpad edema measured at indicated time periods following ET injection (0.15 μg ET/paw). Each data point represents the average of three measurements taken for a single footpad for each mouse. Error bar indicates the standard deviation. Statistical significance was evaluated using an unpaired Student’s t test and represented using standard symbolism (∗∗∗∗*p* < 0.0001, ∗∗*p* < 0.01). (D) Summary diagram of drugs suppressing ET-induced edema identified in this (red text) and prior studies (gray text), and their specific cellular targets mediating ET cellular toxicity.

ET is a major factor contributing to endothelial barrier disruption, leading to tissue edema and cardiovascular collapse, during late stage anthrax infection.^12^^,^^14^^,^^20^ In a previous study, we provided the *in vivo* validation of the therapeutic potential of agents inhibiting cAMP-effectors (e.g., the EPAC inhibitor ESI09 and SecinH3, which blocks the downstream Arf6 GTPase) and the IGF1R inhibitor AG1024 in protecting against ET-induced edema using an *in vivo* quantitative mouse footpad assay.^22^ Results from the current study also indicate essential contributions of Rac1, PI3K, and MEK signaling in mediating the effects of ET on the rearrangement of the F-actin network and barrier destabilization. We therefore tested whether the pharmacological inhibition of these latter components similarly counteracted the robust edema induced by the intradermal injection of ET in mouse footpads (see experimental scheme in Figure 6A, and quantifications in Figure 6B showing a 2-fold increase in thickness of the foot, comparable to the 2– to 3-fold increase observed in a *B.a* infection model^65^). Mice pre-treated with PI3K inhibitor GDC-0941 and MEK inhibitor AS-703026 blocking the early cAMP independent effects of ET or co-treated with the Rac1 inhibitor NSC23766, which acts later in a cAMP dependent fashion, displayed significant reductions in ET-induced edema (Figures 6B and 6C). This pharmacological blockade of ET-induced edema was strongest with the PI3K and Rac1 inhibitors (GDC-0941 and NSC23766, respectively), which provided protection on par with that reported for neutralizing antibody treatments^66^ and was much more effective than treatment with adefovir or other small molecule inhibitors of EF activity (M. Moayeri, unpublished observations). Additionally, all three inhibitors provided prolonged protection against ET-induced edema, which persisted for up to 18 h in these assays. None of the mice pre-treated with GDC-0941 or AS-703026 alone showed any obvious signs of malaise during these experiments, suggesting that these compounds or closely related derivatives may offer potential for future clinical applications. These results connect the molecular response to both cAMP-dependent and independent mechanisms of ET intoxication (Rac1-dependent actin remodeling and RTK activation) to its overt physiological consequences in an *in vivo* model relevant to anthrax infection (see Figure 6D for a summary of pharmacological compounds blocking ET-induced edema in this and previous studies).

## Discussion

This study characterizes cooperative activities of anthrax ET, which disrupts the F-actin network, functioning via both an early acting cAMP-independent activation of RTK signaling and a later cAMP-dependent activation of the Rac1-cofilin axis, in compromising endothelial integrity. We also demonstrate a remarkable measure of protection against ET-induced edema by the chemical inhibition of these pathways. These findings thus open potential therapeutic avenues for blocking intracellular activities of ET during anthrax infection.

Anthrax toxins promote vascular leakage by disrupting endothelial barriers, resulting in severe edema, hypovolemic shock and cardiovascular collapse. Both ET and LT compromise host barrier integrity and induce vascular shock, albeit with distinct pathological consequences.^12^^,^^22^^,^^23^^,^^31^^,^^67^^,^^68^^,^^69^ There is strong evidence that vascular dysfunction during the fulminant stages of infection plays a central role in the fatal outcome of systemic anthrax infection, despite antibiotic-induced bacterial clearance.^70^ The role of anthrax toxemia in disease progression and host lethality is widely agreed upon, acknowledging that the individual and combinatorial involvements of ET and LT in this process are complex.^31^^,^^67^^,^^69^^,^^71^ ET plays a key role in bacterial dissemination and pathological outcomes, particularly at later stages in a mouse model of inhalational anthrax.^72^ Notably, while an ET-deficient mutant (ΔET) strain of anthrax induced significantly less vascular leakage than the WT parent strain, the effect of lethal factor deletion (ΔLF) was much less pronounced, indicating a primary role of ET in endothelial permeability.^23^ Pharmacological measures mitigating the effects of ET could thus effectively support patient survival during advanced, systemic stages of the disease.

Here we show that ET disrupts the actin cytoskeletal network, resulting in the loss of endothelial barrier integrity. The actin cytoskeleton is tightly linked with several vital cellular processes, including motility, trafficking,^73^^,^^74^ and adhesion.^75^^,^^76^^,^^77^ Other pathogenic effectors similarly remodel the cytoskeleton to promote barrier destabilization and host invasion.^78^^,^^79^^,^^80^^,^^81^^,^^82^ Actin remodeling in response to ET, also occurs in several murine and human cell types, including fibroblasts and kidney cells,^83^ macrophages,^84^^,^^85^^,^^86^ and HMVECs,^87^ altering morphology, immune functions, and chemotaxis.^88^

In this study, we identify the small GTPase Rac1 as a central regulator of ET-dependent actin remodeling. Pharmacological inhibition of Rac1 robustly rescued actin rearrangement and permeability induced by ET *in vitro* and *in vivo*. Rac1 regulates barrier integrity through several different effectors. In normal physiological contexts, Rac1 activity is typically regarded as “barrier protective,”^89^^,^^90^^,^^91^^,^^92^^,^^93^^,^^94^^,^^95^ primarily through its activation by cAMP/Epac/Rap1 signaling. However, barrier disruptive effects of Rac1 have also been reported. For example, Rac1-dependent NOX1 activation mediates epithelial barrier dysfunction during rhinovirus (RV) infection.^96^ Rac1 activity also plays a role in endothelial permeability induced by the platelet-activating factor (PAF),^97^ PI3K,^98^ and epithelial barrier disruption by *Salmonella enterica* serovar Typhimurium.^99^ Thus, the cellular mechanism involved in ET-mediated barrier dysfunction is likely to involve a pathological overstimulation of Epac and Rac1 signaling, leading to outcomes opposite to their physiological barrier protective functions, the so-called cAMP paradox.^12^^,^^100^

The role of Rac1 in modulating actin dynamics in many cell types, including endothelial cells and the vasculature, has been extensively characterized.^36^^,^^101^^,^^102^ Rac1 regulates actin polymerization through downstream effectors such as the actin severing protein cofilin (CFL1).^103^ Consistent with Rac1 mediating the disruptive effects of ET on the F-actin network and intercellular barrier, we observed Rac1-dependent activation (dephosphorylation) of CFL1 and its regulatory phosphatase SSH1, which specifically recognizes CFL1 phosphorylated at Ser-3.^104^ A causal relationship between Rac1, and the cofilin-phosphatase activity of SSH1 has been proposed in studies investigating keratinocyte migration^40^^,^^43^ neuronal degeneration^105^ and maturation,^106^ as well as synaptic response to stress in hippocampus and amygdala.^107^ In addition to modulating actin dynamics via the regulation of CFL1 activity, Rac1 can also impact actin polymerization more directly by activating the WAVE regulatory complex (WRC), resulting in the subsequent activation of the Arp2/3 complex and its promotion of F-actin branching and nucleation.^108^^,^^109^^,^^110^ Although we report a causal relationship between Rac1 activity, CFL1 dephosphorylation, and cellular morphological changes resulting from actin rearrangements, a possible CFL1-independent effect of Rac1 on actin dynamics resulting from the activation of nucleation promoting factors such as WAVE merits further examination.

Interestingly, we found that Rac1 inhibition was also protective against another activity of ET: blockade of Sec15-containing exocyst assembly (Figure 3C).^22^^,^^23^ Sec15 complexes assemble in a RalA and Rab11-dependent fashion and ensure proper transit of cargo-loaded vesicles along radial F-actin cables, to deliver junctional components at points of cell-cell contact.^47^^,^^111^ In addition, exocyst complexes have been found to bind to a negative Rac1 regulator (the RacGAP SH3BP1) and to the major WRC effector of Rac1 to control cell migration, suggesting a functional connection between Rac1 and the exocyst.^34^^,^^112^^,^^113^ Thus, the pathological stimulation of Rac1 by ET may influence more than just F-actin dynamics, also deregulating exocyst function to weaken intercellular junctions. These considerations place Rac1 in a central position in the cellular response to cAMP overload induced by ET. CDC42, another GTPase from the Rho subfamily, has also relevance for the regulation of the endothelial barrier,^114^^,^^115^ and is regulated by cAMP-dependent processes.^116^^,^^117^ Whether CDC42 is also implicated in ET-induced F-actin reorganization and edema calls for further investigation.

Interestingly, a recent study shows that prolonged cAMP production induced by ET leads to ATP depletion, which could be responsible -at least in part- for ET-induced footpad edema and lethality.^118^ These two proposed mechanisms, Epac activation versus ATP depletion, may collaborate to mediate different aspects of ET-induced toxemia relevant to moderate versus high toxin doses, respectively.

Our investigations also identified an unexpected role of IGF1R/PI3K/AKT and EGFR/MEK/ERK signaling in Rac1/CFL1-depedent actin remodeling, monolayer permeability, and edema. Thus, ET, ET^mut^, or PA alone can induce the early phosphorylation of both IGF1R and EGFR, implying an early acting cAMP-independent process, in which the receptor may be activated through a non-canonical mechanism known as “transactivation.” IGF1R transactivation has been reported during various infectious and inflammatory processes, such as oncogenic transformation,^119^ colonic inflammation, IBD^120^ and crosstalk with GPCRs to regulate neuronal survival.^121^ Regarding EGFR signaling, pathogen-induced transactivation has been reported for *Streptococcus suis*,^122^ *Helicobacter pylori*,^123^ *Pseudomonas aeruginosa*,^124^ *Chlamydia trachomatis*,^125^ *Klebsiella pneumoniae*,^126^ and *Neisseria gonorrhoeae*.^127^ EGFR transactivation is utilized by these pathogens for various aspects of the infectious process, including pathogen attachment and internalization, epithelial cell scattering, the inhibition of apoptosis and cytoskeletal rearrangements.^56^ Some of these studies have highlighted the role of EGF ligand shedding by matrix metalloproteases (MMPs),^122^^,^^127^ while others indicate a ligand-independent mechanism involving direct EGFR activation by Src-kinases,^126^ or p38 MAPK.^123^ In our study, ET-mediated EGFR transactivation was reduced by both MMP and Src inhibition, suggesting a ligand-dependent transactivation mechanism, but not ruling out the possibility that the Src kinase may also provide direct EGFR activation. Src activation by PA is also important for toxin entry,^64^ raising the possibility that subsequent EGFR activation may also play a role in this process. Interestingly, anthrax lethal factor (LF) inhibits MEK signaling through proteolysis and inactivation of mitogen-activated protein kinase kinases (MAPKK)^19^^,^^128^ and also inhibits the PI3K pathway through cleavage of p85 regulators.^129^ How EF and LF exert opposing effects on MEK and PI3K signaling, and through what integrated mechanism EGFR and IGF1R transactivation contribute to anthrax pathogenesis, will require further investigation.

In summary, this study has identified a set of druggable cellular targets, the inhibition of which effectively mitigates the effects of ET *in vitro* and *in vivo*. A substantial advantage of these compounds is that they may counteract ET toxicity after (or at) the point of host cell entry. In contrast, anti-EF and anti-PA antibodies can only neutralize toxins prior to cell entry.^66^^,^^130^ Moreover, several of these compounds are well-tolerated *in vivo*. These findings open the door to identifying related structural derivatives of these safe and effective compounds for preclinical testing and analysis of additional inhibitors targeting RTK and Rac1 signaling that have been identified in recent years.^131^ Such compounds could offer great promise for treating edema and lethality suppression in the context of toxin challenge and whole animal infection.^132^ Because the effects of LT can be attenuated with factors restoring the function of the ERK pathway,^133^ however, MEK inhibitors may represent a less promising lead than PI3K and Rac1 inhibitors for the treatment of *B.a* infections. Rac1 inhibitors may also be used to treat symptoms caused by other bacterial toxins that either directly or indirectly induce cAMP production, such as Ctx,^24^ Cya,^134^ PTX,^135^ ExoY^136^ that also disrupt the F-actin network. Likewise, PI3K and MEK inhibitors might be repurposed to treat infections by other bacteria that subvert RTK signaling to mediate various pathogenic processes.^56^ With the growing threat of antibiotic resistance, such adjunctive therapies, mitigating the impact of bacterial toxins on host cells and tissues, and less likely to induce the selection of resistant bacterial strains,^137^ are more needed than ever.

### Limitations of the study

In this study, we evaluate how anthrax Edema Toxin (ET) impacts the F-actin cytoskeleton and show that ET activates the small GTPase Rac1 while inhibiting RhoA. Consistent with the hypothesis that Rac1 activation mediates ET effects on F-actin rearrangement, the Rac1 inhibitor NSC23766 blocks all ET-induced phenotypes in cultured endothelial cells and *in vivo*. Additional experimental approaches, such as the blocking expression of Rac1 (using siRNA or shRNA), would be expected to further solidify these conclusions. Sustained Rac1 activation could also result in its degradation, although the *in vivo* data (in which NSC23766 blocks ET-induced edema at 6 h and 18 h) favor the Rac1 activation model. Measuring the total levels of Rac1 in response to ET over an extended time could provide a separate experimental angle on this question. In contrast to Rac1 and RhoA, the potential contribution of other small GTPases of the Rho sub-family -such as CDC42- has not been examined in this study. Chemical inhibition of CDC42 (or blocking its expression) could also be used to evaluate whether CDC42 is also implicated in ET-induced F-actin rearrangements. We note that all experiments in the current study involve purified factors (ET^wt^, ET^mut^, and PA) administered to cultured cells or mouse footpads, not infections with *B.a*. Although the study identifies several small molecule inhibitors that successfully block toxin-induced cytoskeletal disorganization and *in vivo* foot pad edema, it does not evaluate whether these compounds can effectively protect cells and animals from lethal *B.a* infection. Because *B.a* secretes another toxin (LT), and triggers various immunological responses and inflammation, complex cross-talk between different signaling pathways could be at play in response to *B.a* infection. For example, MEK inhibition blocks ET-induced toxicity (this study), but could enhance toxicity by LT, which inhibits MAPK signaling. Future infection experiments (*in vitro* and *in vivo*) should establish the respective clinical value of each identified compound.

## Resource availability

### Lead contact

Further information and requests for resources and reagents should be directed to and will be fulfilled by the Lead Contact, Ethan Bier (ebier@ucsd.edu).

### Materials availability

This study did not generate new unique reagents.

### Data and code availability

• All data reported in this study are available from the lead contact upon request. Raw data have been deposited at Mendeley Data (https://data.mendeley.com/datasets/g5trm4zx6r/1).
• This article does not report original code.
• Any additional information required to reanalyze the data reported in this article is available from the lead contact upon request.

## Acknowledgments

We are grateful to Susanne Heynen-Genel and Debbie Chen (Conrad Prebys Center for Chemical Genomics (CPCCG) High-Content Screening Facility, Sanford Burnham Prebys Medical Discovery Institute), Jennifer Santini and Marcella Erb (Microscopy core, UC-San Diego, supported by grant 10.13039/100000065NINDS P30NS047101) for assistance in microscopy and image data analysis. We thank Danielle O’Mard for assistance with animal studies. We thank Victor Nizet for his thoughtful comments on the article. This research was supported by RO1 AI110713 to Ethan Bier and Victor Nizet, RO1 GM144608, RO1 GM117321, R35 GM1518085, RO1 AI16291 to Ethan Bier, and by the Intramural Program of the 10.13039/100000060National Institute of Allergy and Infectious Diseases, NIH.

## Author contributions

Conceptualization, P.J., A.G., and E.B.; methodology, P.J., M.M., A.G., MF, IR, and E.B.; investigation, P.J., AG, SK, MF, IR, and M.M.; writing-original draft, P.J. and A.G; writing-review and editing, A.G., P.J., M.M., S.H.L., and E.B.; funding acquisition, E.B.; resources, S.H.L. and E.B.; supervision, E.B.

## Declaration of interests

Ethan Bier and Prashant Jain are listed as inventors on a pending U.S. patent Application No.: 17/602,700 entitled “PHARMACOLOGICAL MITIGATION OF LATE-STAGE TOXEMIA” submitted by the University of California, San Diego.

## STAR★Methods

### Key resources table

**Table:** 

REAGENT or RESOURCE	SOURCE	IDENTIFIER
**Antibodies**		

Rabbit anti-Akt	Cell Signaling Technology	Cat# 9272; RRID: AB_329827
Rabbit anti-Cofilin	Cell Signaling Technology	Cat# 3318; RRID: AB_2080595
Rabbit anti-EGF Receptor	Cell Signaling Technology	Cat# 4405; RRID: AB_331380
Rabbit anti-IGF-I Receptor β	Cell Signaling Technology	Cat# 3027; RRID: AB_2122378
Rabbit anti-p44/42 MAPK (ERK1/2)	Cell Signaling Technology	Cat# 9102; RRID: AB_330744
Rabbit anti-Phospho Cofilin (Ser3)	Cell Signaling Technology	Cat# 3311; RRID: AB_330238
Rabbit anti-Phospho-Akt (Ser473)	Cell Signaling Technology	Cat# 4060; RRID: AB_2315049
Rabbit anti-Phospho-EGF Receptor (Tyr1068)	Cell Signaling Technology	Cat# 2234; RRID: AB_331701
Rabbit anti-Phospho-IGF-I Receptor β (Tyr1135/1136)	Cell Signaling Technology	Cat# 3024; RRID: AB_331253
Rabbit anti-Phospho-p44/42 MAPK (ERK1/2)	Cell Signaling Technology	Cat# 9101; RRID: AB_331646
Rabbit anti-Phospho-slingshot-1L (Ser978)	ECM Biosciences	Cat# SP3901; RRID: AB_10553849
Rabbit anti-Slingshot-1L	ECM Biosciences	Cat# SP1711; RRID: AB_2195478

**Chemicals, peptides, and recombinant proteins**		

AS-703026 (MEK inhibitor)	Selleckchem	Cat# S1475
Batimastat (MMP inhibitor)	Selleckchem	Cat# S7155
GDC-0941 (PI3K inhibitor)	Selleckchem	Cat# S1065
HCS CellMask green dye	Thermo Fisher	Cat# H32714
Lipofectamine 3000 Transfection Reagent	Thermo Fisher	Cat# L3000001
NSC23766 (Rac1 inhibitor)	Tocris	Cat# 2161
PP2 (Src-kinase inhibitor)	Selleckchem	Cat# S7008
ProLong Gold Antifade Mountant	Thermo Fisher	Cat# P36930
Protease inhibitor cocktail	Millipore Sigma	Cat# 11697498001
RIPA lysis buffer containing phosphatase inhibitor (PhosSTOP)	Millipore Sigma	Cat# 4906845001
RPMI 1640 media	Corning	Cat# 10-104-CV
Alexa 488-Phalloidin	Thermo Fisher	Cat# A12379
Alexa 568-Phalloidin	Thermo Fisher	Cat# A12380

**Critical commercial assays**		

Rac1 pull-down activation assay biochem kit	Cytoskeleton Inc.	Cat# BK035
RhoA pull-down activation assay biochem kit	Cytoskeleton Inc.	Cat# BK036

**Deposited data**		

Raw images and quantifications	This study	https://data.mendeley.com/datasets/g5trm4zx6r/1https://doi.org/10.17632/g5trm4zx6r.1

**Experimental models: Cell lines**		

Human brain microvascular endothelial cell line (HBMEC)	Provided by V. Nizet, UCSD	N/A

**Experimental models: Organisms/strains**		

C57BL/6J mice (8 weeks old males)	The Jackson Laboratory	Cat# 000664; RRID: IMSR_JAX:000664

**Recombinant DNA**		

Sec 15 GFP plasmid	Provided by C. Mitchell, Monash University, Australia	N/A

**Software and algorithms**		

Adobe Illustrator 24.0.3	Adobe Inc.	https://www.adobe.com/products/illustrator.
Adobe Photoshop 24.4.1	Adobe Inc.	https://www.adobe.com/products/photoshop.
Powerpoint 16.102	Microsoft	https://www.microsoft.com/en-us/microsoft-365/powerpoint
GraphPad Prism 10	GraphPad Software, Inc.	https://www.graphpad.com/
ImageJ 2.16.0	N/A	https://imagej.nih.gov/

**Other**		

PET membrane permeable support 1μm pore size	Corning	Cat# 353103

### Experimental model and study participant details

#### Cell lines

Immortalized Human brain microvascular endothelial cells (HBMECs) were obtained from Victor Nizet (UCSD). Cells were not further authenticated nor checked for mycoplasma, as no sign of contamination was apparent.

#### Animal models

C57BL/6J (8 weeks old males) mice used in this study were obtained from Jackson Labs (Bar Harbor, ME). All *in vivo* experiments were performed under protocol LPD-8E approved by the Animal Care and Use Committee of the National Institute of Allergy and Infectious Diseases, National Institutes of Health.

### Method details

#### Toxins

Endotoxin-free preparations of wild-type Edema factor (EF), catalytically inactive EF mutant (EF K313R) and protective antigen (PA) were purified from *B. anthracis* as previously described.^31^^,^^138^ The wild-type and mutant EF used in this work have the N-terminal sequence ANEHYTES for maximum toxin activity.^139^

#### Cell culture, inhibitor treatment and ET intoxication

HBMEC cultures were maintained in RPMI1640 (Corning) containing 10% FBS, 1% penicillin/streptomycin, 2 mM L-glutamine, and were incubated in 37°C, 5% CO_2_ atmosphere. EF and PA were added at a ratio of 1:2 and cells were further incubated for indicated time periods at 37°C, 5% CO_2_ atmosphere. Pharmacological inhibitors of PI3K (GDC-0941, Selleckchem; #S1065), Src-kinase (PP2, Selleckchem; #S7008), MMP (Batimastat, Selleckchem; #S7155) and Rac1 (NSC23766, Tocris; #2161) were added to cells 1 h prior to EF/PA treatment, while the MEK inhibitor (AS-703026) (Selleckchem, #S1475) was added to cells for overnight and then 1 h prior to EF/PA treatment.

#### Analysis of F-actin and Sec15GFP dots distribution

HBMECs were cultured to ∼60% confluence on glass poly-D-lysine coated chamber slides (BD Falcon #354108) for all experiments, except for Figure 1, where cells were seeded on collagen-coated coverslips and 33 ng/ml hydrocortisone was added to the medium. Cells were treated with EF+PA in the presence and absence of inhibitors 4 h before fixation (4% paraformaldehyde in PBS for 30 min at room temperature). Cells were permeabilized in 0.25% Triton-X 100 in PBS for 5 min at 4°C and incubated with phalloidin conjugated with Alexa fluor 488 or 568 (ThermoFisher) overnight at 4°C. Slides were mounted with Prolong Gold with DAPI mounting media (ThermoFisher) and cured overnight at room temperature. For the analysis of Sec15-GFP aggregates distribution, cells were grown at ∼80% confluence and transfected with Sec15GFP plasmid, using Lipofectamine 3000 transfection reagent (Life Technology Inc.) per manufacturer’s instructions. EF/PA and inhibitor treatments were performed 48 h post-transfection. Cells were fixed in 4% paraformaldehyde in PBS for at least 30 min in room temperature. Slides were mounted with Prolong Gold with DAPI mounting media and cured for overnight at room temperature. Fluorescence images of F-actin and Sec15-GFP were acquired using a confocal microscope Leica SP8 (Figure 1) or a Delta Vision RT (Applied Precision) microscope (Figures 2, 3, 4, and 5).

#### Actin fiber quantifications

Raw images were analyzed in ImageJ. Actin fibers were quantified using the Biovoxxel Plugin, following the steps (See Figures 1B and S1):

1. File>Open Select .tif image to open.
2. Image>Type>8-Bit.
3. Process>Binary>Options make sure black background is checked>Ok.
4. Image>Adjust>Threshold (parameters: 150 to 255) Apply and Set. Dark background should be selected in the thresholding window.
5. Process>Binary>Convert to Mask.
6. Plugins>Biovoxxel>Extended Particle Analyzer (area: 0.5-infinity; circularity 0-0.5; aspect ratio 7-infinity; show masks; display results).

For F-actin phenotypes in Figure 2C, the thresholding parameters were 55 to 255, and aspect ratio values were 5-infinity.

#### High content, medium-throughput cell-size quantification

HBMECs were seeded in 96-well clear bottom plates-black (Corning; #3603) at a final confluency of approximately 70%. For evaluating the effect of pharmacological inhibitors, cells were pretreated with the Rac1 inhibitor NSC23766 and IGF1R inhibitor AG1024 for 1 h and then treated with 250 ng/ml ET for 4 h. The cells were washed 3 times with PBS and fixed in 4% paraformaldehyde prepared in PBS for 30 min in room temperature. The cells were washed 3 times with PBS and incubated with PBS containing DAPI with HCS CellMask Green dye (ThermoFisher Scientific; #H32714) to uniformly stain the cytoplasm and nucleus, per manufacturer’s protocol. All treatments and washes were performed manually using a multi-channel pipette to maintain consistency between treatments. Columns 1 and 12 of each plate were left untreated and stained with DAPI in PBS alone to serve as ‘blank or background’ controls for high-content image analysis. The cells were washed 3 times with PBS and imaged using Opera QEHS high-content screening system (PerkinElmer). Automated analysis of cell size was conducted using a custom script. *p* values were calculated by unpaired Student t test using GraphPad Prism software.

#### Rac1-GTP and RhoA-GTP pull-down assay

Rac1-GTP and RhoA-GTP pull-down assays were performed using the Rac1 pull-down activation assay biochem kit (Cytoskeleton Inc.; #BK035) and RhoA pull-down activation assay biochem kit (Cytoskeleton Inc.; #BK036). Briefly, HBMEC were seeded in 12-well tissue culture plates at an overnight confluency of approximately 60%. Following overnight incubation at 37°C, 5% CO_2_ atmosphere, media in each well was replaced with fresh media containing 1% FBS. After 24 h incubation, cells were treated with 250 ng/ml ET for 2 h or 4 h in the presence or absence of indicated inhibitor. Whole-cell lysates were prepared according to manufacturer’s instructions. Approximately 600 μg of whole-cell lysates was incubated on a rotator for 1 h at 4°C, with PAK-PBD beads for Rac1-GTP pull down, or with Rhotekin-RBD beads for RhoA-GTP pull down and processed for western blot analysis per manufacturer’s protocol.

#### Immunoblotting

80% confluent cells were lysed in RIPA lysis buffer containing phosphatase inhibitor (PhosSTOP) and protease inhibitor cocktail, from Sigma-Aldrich. Lysates were sonicated and clarified at 10,000 g for 10 min at 4°C. 20 μg total protein per sample was resolved in SDS-PAGE. Proteins were subsequently transferred to PVDF membrane and blocked with 3% BSA in TBS containing 0.1% Tween 20 (TBS-T). Blots were incubated with primary antibodies diluted in TBS-T for overnight at 4°C. Primary antibodies for pIGF1Rβ (#3024S), IGF1Rβ (#3027S), pEGFR (#2234S), EGFR (#4405S), pAKT (#4060S), AKT (#9272S), pERK (#9101S), ERK (#9102S), pCFL1 (#3311S) and CFL1 (#3318S), were all purchased from Cell Signaling Technology (Danvers, MA). Antibodies against pSSH1 (#SP3901) and SSH1 (#SP1711) were purchased from ECM Biosciences (Versailles, KY), and Rac1 antibody was included in the Rac1 pull-down activation assay biochem kit (Cytoskeleton Inc.). Blots were washed with TBS-T and probed with species-specific HRP-conjugated secondary antibodies (Bio-Rad).

#### Transwell permeability assay

HBMECs were seeded on transwell PET membrane permeable support- 1 μm pore size (Corning; #353103) placed in 12-well companion plates (Corning; #353503). The insert (luminal compartment) contained 0.5 ml media, while the lower chamber (albuminal compartment) contained 1.5 ml media. Cells were cultured at an initial confluency of less than 30% and incubated in 37°C, 5% CO_2_ atmosphere for 7 days to full confluency. Media from apical and basal chamber was replaced every 2 days, till a confluent monolayer was formed. Media in the apical chamber was replaced with fresh media containing inhibitors. Cells were incubated with the Rac1 inhibitor NSC23766 for 1 h prior to the addition of ET and Evans blue-albumin (0.335 mg/ml Evans blue, 2 % BSA in PBS). Total volume of the luminal chamber was maintained at 0.5 ml. Media in the basal chamber was replaced, after two washes with PBS, with 1.5 ml permeability assay buffer (141 mM NaCl, 2.8 mM CaCl_2_, 1 mM MgSO_4_, 4 mM KCl, 1 mM NaH_2_PO_4_, 10 mM Glucose, 10 mM HEPES pH 7.4) as reported previously.^140^ After 24 h, assay buffer from the albuminal chamber was evenly mixed by pipetting, and 100 μl sample was transferred to a 96-well clear plate (Greiner Bio-One). The Evans blue-albumin concentration in the sample was determined by measuring the absorbance at 630 nm with a SpectraMax Plus microplate reader (Molecular Devices).

#### *In-vivo* mouse footpad edema assay

C57BL/6J mice (8 weeks old males, n=5/group, Jackson Labs, Bar Harbor, ME) were treated with PI3K inhibitor GDC-0941 (75 mg/kg) or MEK inhibitor AS-703026 (33 mg/kg), by gavage (200 μl/administration), at 18 and 4 h prior to toxin administration. Control animals were administered with the same volume of vehicle (4% DMSO+ 30% PEG300+ 5% Tween 80 in ddH20). ET was injected intradermally into a single footpad (0.15 μg/20 μl) and PBS (20 μl) was injected in the opposing paw footpad. Rac1 inhibitor NSC23766 (10 mg/kg) was co-administered into footpads with the ET, and PBS was used as the vehicle control for this drug in the opposing footpad. Three footpad measurements were taken in the dorsal/plantar direction using digital calipers at 8 h and 18 h post-toxin administration.

### Quantification and statistical analysis

Statistical significance was calculated in GraphPad Prism (version 7.0d) using unpaired Student’s t-test. Standard symbolism was used for statistical significance: ∗∗∗∗*p*<0.0001, ∗∗∗p<0.001 ∗∗*p*<0.01, ∗*p*<0.05, ns: non significant.
