# Supplementary material for: Anthrax ET activates Rac1 and RTK signaling to induce F-actin reorganization and endothelial permeability
Source: iScience. 2025 Oct 3;28(11):113682. doi: 10.1016/j.isci.2025.113682 (PMC12555787; doi:10.1016/j.isci.2025.113682)
Supplement: Document S1. Figures S1–S4 [file mmc1.pdf]

iScience, Volume *28*

Supplemental information

Anthrax ET activates Rac1 and RTK signaling to induce F-actin reorganization and endothelial permeability

Prashant Jain, Annabel Guichard, Mahtab Moayeri, Saluja Kaduwal, Margot Mel de Fontenay, Ian Rousseau, Stephen H. Leppla, and Ethan Bier

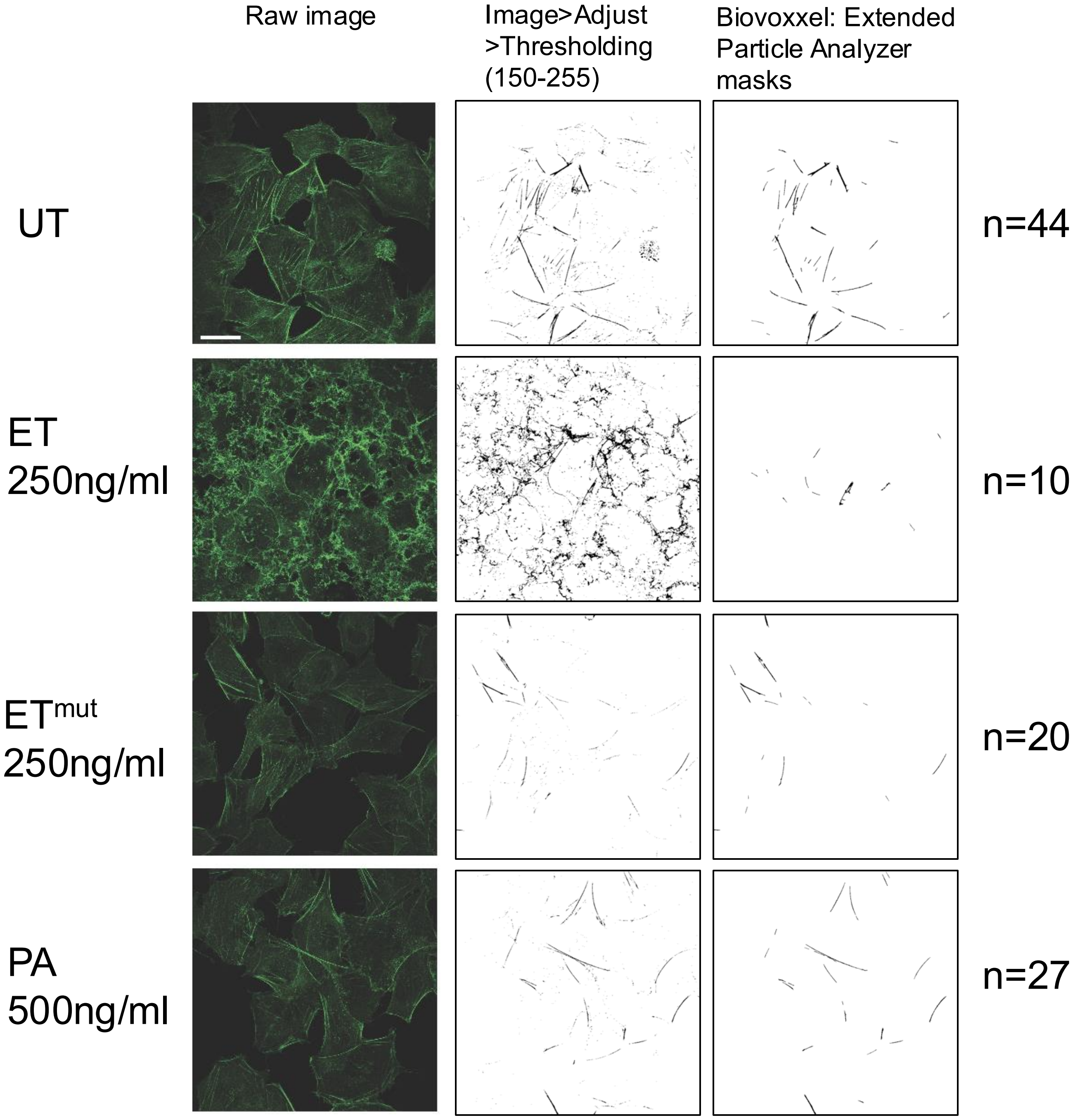

Figure S1. Actin stress fiber quantifications using ImageJ. Confocal images were opened in ImageJ (left images), F-actin fibers masks and numbers were obtained using the following steps (also described in the methods section): 1) File>Open Select .tif image to open. 2) Image>Type>8-Bit. 3) Process>Binary>Options make sure black background is checked>Ok. 4) Image>Adjust>Threshold (150 to 255) Apply and Set. Dark background should be selected in the thresholding window. 5) Process>Binary>Convert to Mask Plugins>Biovoxxel>Extended Particle Analyzer (area: 0.5-infinity;circularity 0-0.5; aspect ratio 7-infinity; show masks; display results). ET treatment disrupts cytoskeletal organization and severly reduces the number of F-actin fibers, while ET^{mut} and PA cause milder phenotypes, mainly reducing the number of actin fibers, without altering the whole cytoskeletal organization.

A F-actin intensity profiles

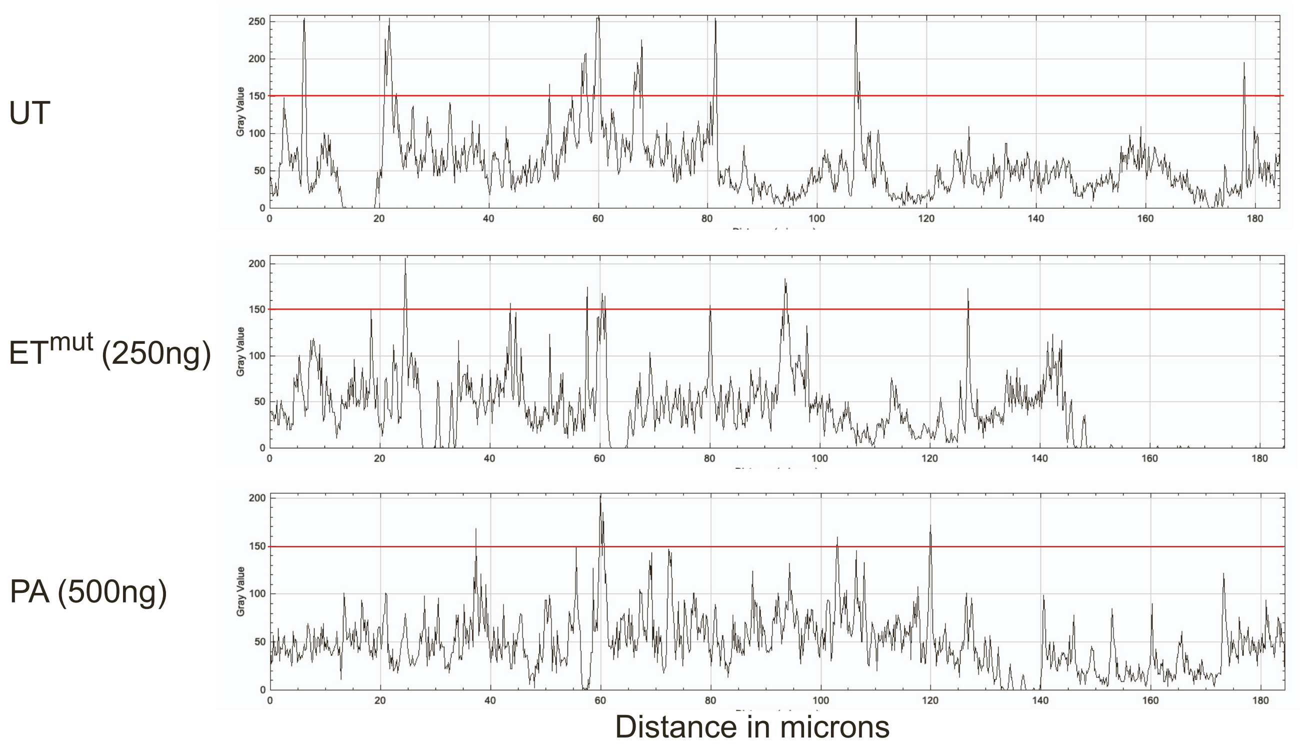

B F-actin intensity profile quantifications

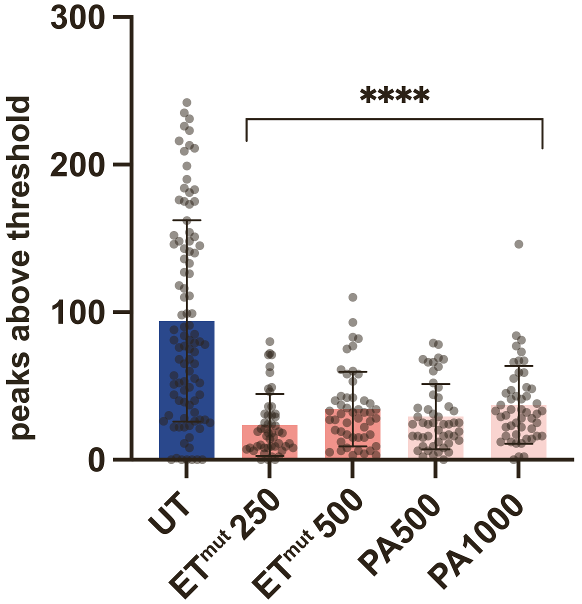

Figure S2: F-actin intensity profiles reveal the effects of ETmut and PA. **(A)** Representative examples of intensity plot for F-actin images shown in Figure 1A. Cortical actin and sress fibers result in high intensity peaks in untreated cells, which are reduced by ET^{mut} and PA. **(B)** Numers of peaks above the 150 threshold per line have been plotted for each condition using the followng steps: 1) Draw a line across the image. 2) Analyze>Plot Profile. 3) Click Data>Copy All Data Data was pasted into a google sheet. 10 evenly spaced intensity profiles were collected per image. The number of peaks above a given threshold was used using the following code: =COUNTIF(Sheet1!JU2:Sheet1!JU1025,">"&A1) - Where Sheet 1 contains the raw intensity data and A1 contains the threshold. Unpaired Student t-test was used to evaluate statisical significance (****p<0.0001). ET^{mut} and PA reduce significantly the number of peaks above the threshold.

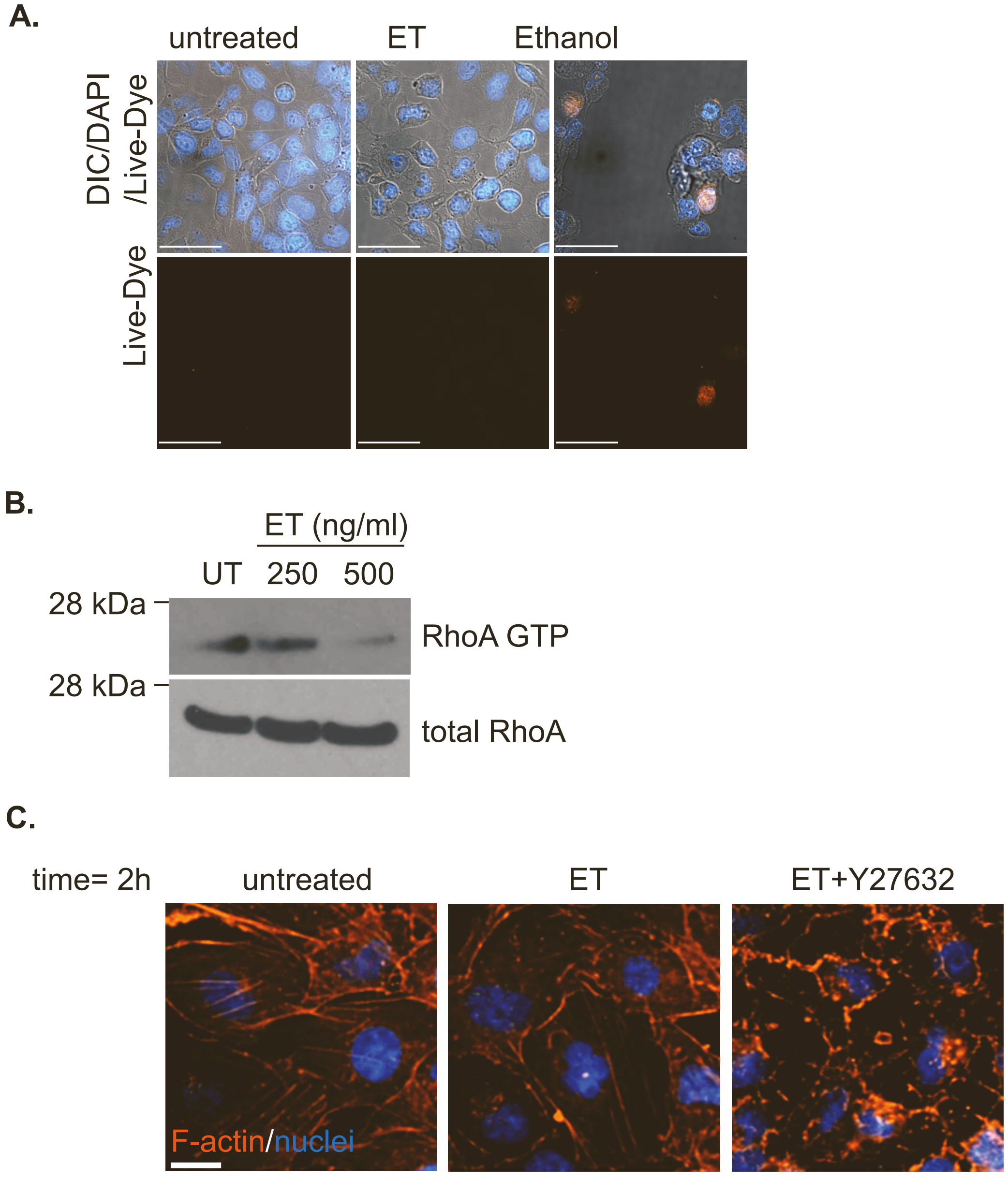

Figure S3. ET does not induce cell death and decreases RhoA activity in HBMECs. **(A)** HBMEC were treated with ET (500 ng/ml) for 4 h, or with 15% ethanol for 10 min to induce cell membrane permeabilization. Cells were stained with DAPI and with the fixable viability dye, Live-Dye 568 to visualize dead cells. Scale bars represent 40 μm. **(B)** HBMEC were treated with 250 and 500 ng/ml of ET for 4 h. Whole-cell lysates were incubated with Rhotekin RBD beads, which specifically bind to GTP-bound RhoA. Bead-bound RhoA-GTP and total RhoA levels in cell lysate were determined by western blot with anti-RhoA antibody. See Figure 2B for analysis of Rac1-GTP levels post-ET treatment.**(C)** HBMEC were treated with 250 ng/ml ET for 2 h, in the presence or absence of Y27632, an inhibitor of the RhoA downstream effector ROCK, and stained with Alexa Fluor 488-conjugated phalloidin to visualize F-actin. Scale bar represents10μm.

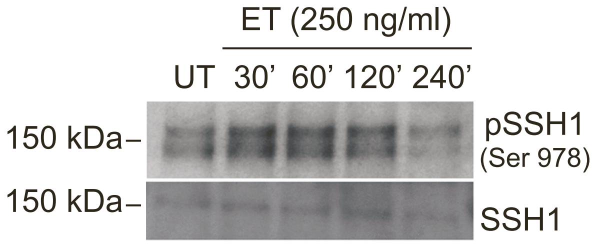

Figure S4. ET activates SSH1. HBMECs were treated with 250 ng/ml of ET for the indicated time periods. Activation (dephosphorylation) of SSH1 in ET-treated and control cells was assessed by monitoring the levels of SSH1 phosphorylated at Serine 978 relative to total SSH1 in whole-cell lysates by western blot. SSH1 activation is detected after 2-4h of treatment.
